# Lithography-Free Route to Hierarchical Structuring of High-χ Block Copolymers on a Gradient Patterned Surface

**DOI:** 10.3390/ma13020304

**Published:** 2020-01-09

**Authors:** Ha Ryeong Cho, Ayoung Choe, Woon Ik Park, Hyunhyub Ko, Myunghwan Byun

**Affiliations:** 1 Department of Materials Engineering, Keimyung University, Daegu 42601, Korea; haryeungcho@gmail.com; 2 School of Energy and Chemical Engineering, Ulsan National Institute of Science and Technology (UNIST), Ulsan 44919, Korea; ayoung5678@unist.ac.kr; 3 Department of Materials Science and Engineering, Pukyong National University, Pusan 48513, Korea; thane0428@pknu.ac.kr; 4 Department of Advanced Materials Engineering, Keimyung University, Daegu 42601, Korea

**Keywords:** high *χ* di-block copolymer, wedge-on-Si geometry, controlled evaporative self-assembly, hierarchically ordered nanostructures, gradient patterned surface, oxide nanogroove

## Abstract

A chemically defined patterned surface was created via a combined process of controlled evaporative self-assembly of concentric polymer stripes and the selective surface modification of polymer brush. The former process involved physical adsorption of poly (methyl methacrylate) (PMMA) segments into silicon oxide surface, thus forming ultrathin PMMA stripes, whereas the latter process was based on the brush treatment of silicon native oxide surface using a hydroxyl-terminated polystyrene (PS-OH). The resulting alternating PMMA- and PS-rich stripes provided energetically favorable regions for self-assembly of high χ polystyrene-*block*-polydimethylsiloxane (PS-*b*-PDMS) in a simple and facile manner, dispensing the need for conventional lithography techniques. Subsequently, deep reactive ion etching and oxygen plasma treatment enabled the transition of the PDMS blocks into oxidized groove-shaped nanostructures.

## 1. Introduction

Irreversible drying droplets comprising nonvolatile solutes (e.g., polymers, colloids, nanoparticles, proteins, DNA, etc.) and volatile solvents often produce irregular dissipative ring-shaped deposits (i.e., “coffee rings”), fingering instabilities, and polygonal patterns via kinetically trapped self-assemblies [1]. The formation of these spatially organized structures is mainly driven by the duplicative pinning–depinning motions of the contact line, whereby the edge of the drying droplet and the solid surface alternate between sticking to each other (i.e., pinning) and sliding over each other (i.e., depinning) [1,2,3]. The stochastic distribution of the formed structures is originally derived from the lack of control over the pinning–depinning locomotion and the presence of temperature-gradient-induced convection [1,2,3]. Hence, a number of advanced research works have been spawned for achieving the control of drying dynamics essentially required for potential improvement to ink-jet printing [4], pathways to patterning surfaces [5], and techniques for disease detection [6]. Diverse, well-established approaches have emerged to enforce the drying droplets to irreversibly evaporate in restricted areas, such as “curve-on-flat” geometry [7,8,9,10,11,12,13,14], two plates with a tiny gap [15,16,17,18,19,20], and cylindrical tube [21,22,23,24]. These confined geometries benefit the routinization of the pinning–depinning motion and the minimization of convection-mediated instability, thus enhancing the uniformity and fidelity of the self-assembled surface structures. In addition, the regular surface patterns obtained from self-assembly can also serve as guided templates for selective deposition of functional nanoscale elements such as carbon nanotubes, biomolecules, nanoparticles, and so on. 

The self-assembly of diblock copolymer (di-BCP) composed of two chemically incompatible chains covalently linked at one end have gained considerable attention for the past two decades as an unconventional bottom-up approach to create a wide range of spatially-ordered nanoscopic domains, including spheres, cylinders, gyloids, and lamellae, that are strongly depending on the volume fraction of components [25]. The self-assembled di-BCP morphologies with domain size ranging from 10 to 100 nm can be effectively controlled by thermal annealing (TA), or solvent vapor annealing (SVA) (i.e., solvent vapor annealing above room temperature), thereby offering templates and scaffolds for the coordination of nanostructured materials [11,18,26] and devices (e.g., memory [27] and energy harvesting devices [28,29]). Thus, the control over the orientation and lateral ordering of nanoscale blocks is of critical importance in the self-assembly of di-BCPs. To date, various kinds of techniques have been exploited for securing the configurational orientation and ordering of blocks such as mechanical shearing [30], external electric field [31], temperature gradient [32], solvent-vapor annealing [33,34], controlled interfacial interaction [35], topographically [36,37] or chemically patterned surfaces [9,38,39], laser ablation [40,41], and so on. Particularly, among them, the use of topographically and chemically patterned surfaces for direct self-assembly (DSA) of di-BCPs suffers from high processing cost and complicated multi-step procedures due to top-down lithography-based process. Therefore, there is still a challenge to develop a noble and facile strategy for the preparation of chemically modified surfaces for effective DSA of di-BCPs. Recently, we successfully demonstrated hierarchically ordered nanostructures self-assembled on chemically distinct gradient surfaces driven by the unfavorable interfacial–interaction–destabilization of thin films of di-BCP with a low segment–segment interaction parameter, *χ*, (i.e., Flory-Huggins interaction parameter) [9]. However, despite this great effort, a functional high *χ* di-BCP with slow self-assembly kinetics because of the slow chain mobility or diffusivity (i.e., stronger segregation of A-A blocks than A-B blocks) has not much been exploited [40,41,42,43,44].

Herein, we demonstrate a facile and robust route to the creation of hierarchically ordered nanostructures composed of high *χ* di-BCPs by combining two consecutive self-assembly processes at micro- and nanometer scales. At macroscale, polymer stripe patterns with a gradient fashion in stripe thickness, *h*, and width, *w*, as well as the center-to-center spacing between neighboring stripes, *λ*_c-c_, were obtained from controlled evaporative self-assembly (CESA), of the drying solution containing nonvolatile polymer solute and volatile organic solvent in a wedge-on-flat geometry comprising a wedge-shaped lens as the upper surface and a flat Si wafer as the lower surface. Subsequently, nonvolatile polymer segments were physically adsorbed on the Si with ~2 nm-thick native oxide layer via hydrogen linkage after vigorous washing with solvent and further surface treatment with polymer brush, thus serving as a guided template with chemically distinct nature (i.e., brush-treated and physically adsorbed surfaces). A di-BCP thin film spin-coated on top of the chemically modified Si substrate was thermodynamically self-assembled into hierarchically ordered nanostructures via the synergy between preferential segregation of di-BCP thin film into brush-treated regions (i.e., dewetting of di-BCP from physically adsorbed surfaces) at the micrometer scale and the solvent-vapor-mediated microphase separation of di-BCP domains at the nanometer scale. Uncommonly and exceptionally, this approach would be a simple and unconventional platform for the self-assembly of di-BCPs in chemically defined regions, eliminating the need for conventional lithography techniques to prepare chemically patterned surfaces reported in copious previous studies.

## 2. Materials and Methods 

***Wedge-on-Si geometry***: An aluminum wedge lens and a Si wafer with ~2 nm thick native oxide layer were used as the upper slanted and the lower flat surfaces, respectively. Areal dimension of the wedge lens was 1 × 1 cm^2^ and the height was 1 mm. The Si (p-type, <100>) substrate was cleaned using a mixture of sulfuric acid and NOCHROMIX, and then intensively flushed with deionized water and exsiccated with N_2_. The wedge-on-Si geometry was set in a homemade-sealed chamber to minimize the effect of air convection and to keep temperature (~25 °C) constant during the whole process of CESA. 

**Figure 1:**
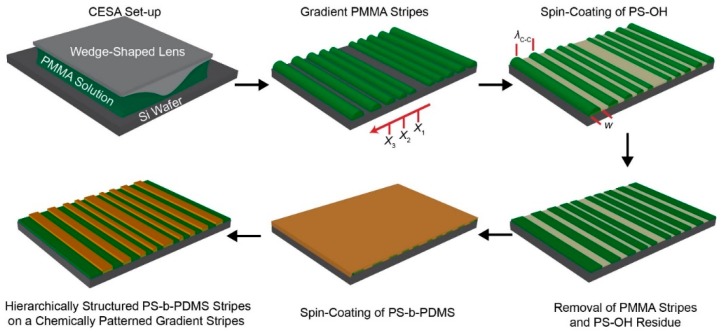
A schematic illustration of formation of hierarchically ordered polystyrene-*block*-polydimethylsiloxane (PS-*b*-PDMS) stripes with a gradient fashion in terms of the stripe width, *w*, and the center-to-center spacing, *λ*_c-c_. Upper left panel: a poly (methyl methacrylate) (PMMA) toluene solution was confined and evaporated in the wedge-on-Si geometry. Upper middle panel: PMMA stripes were produced via controlled evaporative self-assembly (CESA). *X*_n_ (n = 1–3) is the stripe position from the wedge/Si contact center. Upper right panel: hydroxyl-terminated polystyrene (PS-OH) dissolved in 1,2-dichloroethane was spin-coated onto a PMMA patterned sample. Lower right panel: PMMA stripes and PS-OH residue were washed away using toluene after chemical anchoring of PS-OH onto a silicon native oxide surface. Lower middle panel: high χ PS-*b*-PDMS dissolved in toluene was spin-coated and then annealed using toluene vapor at 35 °C. Lower left panel: Surface tension-driven dewetting (i.e., preferential segregation) of the PS-*b*-PDMS thin film led to hierarchically structured PS-*b*-PDMS stripes on chemically defined regions.

***Materials and sample preparation***: Poly (methyl methacrylate) (PMMA, *M*_n_ = 534 kg mol^−1^, *PDI* = 1.57, Sigma-Aldrich) as a nonvolatile solute was dissolved in toluene. The PMMA toluene solution with concentration of 0.5 mg mL^−1^ was trapped in a confined wedge-on-Si geometry and formed a capillary-held solution, allowing the maximum evaporation rate at the perimeter of drying microfluid as schematically illustrated in the upper left panel of Figure 1. Dimensional features including *h*, *w*, and *λ*_c-c_ can be readily tuned by changing the height of the wedge-shaped lens. As the evaporation proceeded in a timely manner, the initial contact angle of the meniscus at the capillary edge gradually decreased due to irreversible evaporative loss of toluene and then reached a critical value, where the nonlinear capillary force (i.e., depinning force) outgrew the linear pinning force, thereby leading to slide of the contact line toward the wedge/Si contact center [45]. Through these repetitive pinning and depinning motions, in overall, concentric gradient stripes within a rectangular area were produced and straight lines remained by cutting the curved regions of the rectangle as shown in the upper middle panel of Figure 1. A hydroxyl-terminated polystyrene (PS-OH, *M*_n_ = 42 kg mol^−1^, PDI = 1.03, Polymer Source) dissolved in 1,2-dichloroethane as a selective solvent for PS-OH, not for PMMA (solution concentration = 20 mg mL^−1^), was spin-coated on to the samples at 3000 rpm for 30 s. Subsequent thermal treatment at the elevated temperature of 150 °C for 2 h triggered chemical anchoring of PS-OH onto a non-patterned Si surface because of condensation reaction between hydroxyl groups of PS-OH and native oxide layer, as schematically illustrated in the upper right panel of Figure 1. The resulting samples were immersed in toluene for removal of the PMMA stripes and residue of PS-OH (lower right panel of Figure 1). A cylinder forming polystyrene-*block*-polydimethylsiloxane (PS-*b*-PDMS, *M*_n_ = 45.5 kg mol^−1^, *PDI* = 1.08, a PDMS fraction = 33.7%, Polymer Source) was selected as a high *χ* di-BCP and dissolved in toluene to prepare the solution with concentration of 10 mg mL^−1^. The PS-*b*-PDMS toluene solution was spin-coated onto the samples and then exposed to toluene vapor for 30 min at an elevated temperature of 35 °C to enhance preferential segregation/microphase separation and to compensate self-assembly kinetics (lower middle and left panels of Figure 1).

***Characterization***: Optical microscopy (OM, BX 51, Olympus, Tokyo, Japan) in reflection mode, atomic force microscopy (AFM, DI3100, Veeco/Digital Instrument/Bruker, Billerica, USA) in tapping mode, and field-emission scanning electron microscopy (FE-SEM, Hitachi, Tokyo, Japan) were applied for characterizing dimensional features including *h*, *w*, and *λ*_c-c_, of the PMMA stripes formed one the flat Si substrate and morphological evolution of the PS-*b*-PDMS thin films before and after solvent vapor annealing using toluene. BS-tap 300 tips (Budget Sensors, Sofia, Bulgaria) with spring constants ranging from 20 to 75 Nm^−1^ were used as scanning probes in a tapping mode. Oxygen plasma treatment (60 Watt and 30 s) and a reactive ion etching (RIE) system using CF_4_ were conducted for oxidization of the PDMS blocks and removal of the top PS layer. X-ray photoelectron spectroscopy (XPS, ESCALAB 250Xi, Thermo Fisher Scientific, Waltham, USA) was utilized for examining the physical adsorption of the PMMA segments on the Si substrate with very thin native oxide layer.

## 3. Results and Discussion

**Figure 2:**
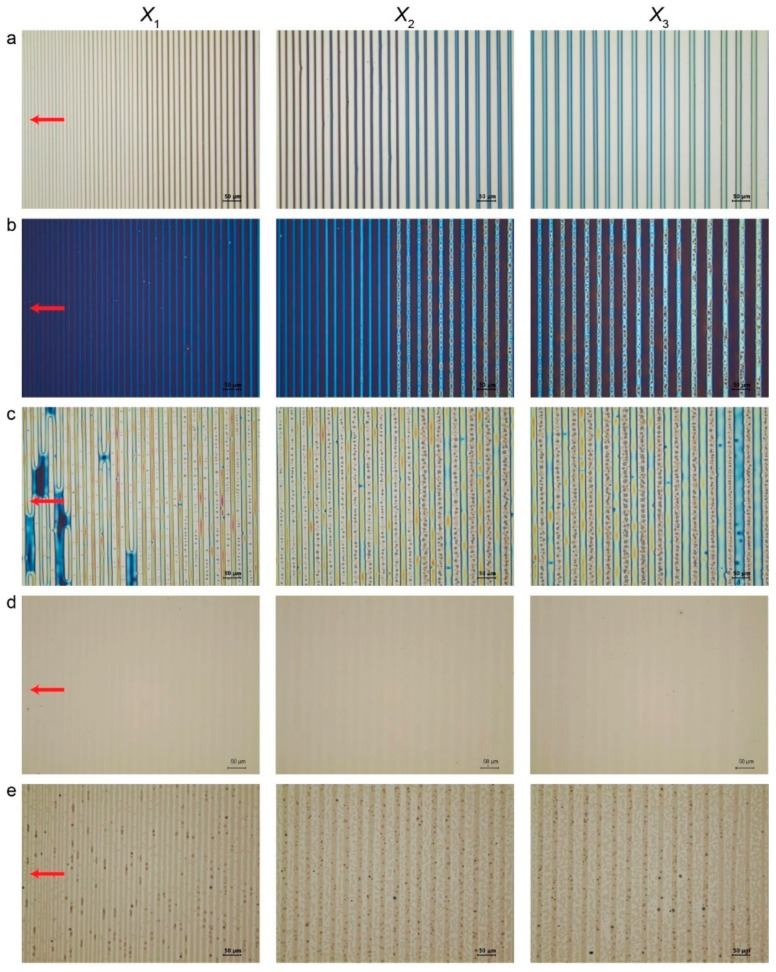
A series of optical micrographs obtained from each step. (**a**) Gradient PMMA stripes right after CESA; (**b**) PS-OH brush spin-coated on to the sample; (**c**) right after chemical anchoring of PS-OH on to a silicon native oxide before washing of PMMA; (**d**) continuous PS-*b*-PDMS thin film spin-coated on to alternating PS-rich and PMMA-rich surface; and (**e**) preferentially segregated PS-*b*-PDMS stripes after solvo-thermal annealing process. The red-colored arrows indicate the moving direction of the three-phase contact line. All scale bars are 50 μm. *X*_n_ (n = 1–3) is the stripe position from the wedge/Si contact center.

As clearly displayed in Figure 2, the PMMA line patterns were observed with OM in reflection mode, appearing in a gradient fashion in terms of *w* and *λ*_c-c_, ranging from 7.2 ± 0.2 μm and 12.4 ± 0.3 μm at the innermost region *X*_1_ to 10.4 ± 0.1 μm and 17.5 ± 0.2 μm at the intermediate region *X*_2_ and to 13.3 ± 0.2 μm and 21 ± 0.3 μm at the outermost region *X*_3_, where *X*_n_ is the distance away from the wedge/Si contact center (upper middle panel of Figure 1 and Figure 2a). The dimensional features of the resulting PMMA stripes can be readily controlled by tuning the height of the wedge lens (i.e., capillary height; vertical distance from the Si substrate) [7]. For the present study, the stripe thickness *h* was not investigated because there was no effect on the whole experimental process. We first examined the physical adsorption of the PMMA segment on the Si substrate. The PMMA stripes were completely removed by vigorously washing with acetone. As previously well-described [9], the PMMA segments were physically adsorbed on the Si substrate with ~2 nm thick native oxide layer via hydrogen bonding between the hydroxyl groups of silicon oxide and the carboxylic groups of the PMMA segments, thereby leading to chemically defined gradient surfaces [7,46]. Thickness of this ultrathin layer was approximately ~2.2 Å (i.e., lengths of hydroxyl and carboxylic chains ≈ 0.97 Å and 1.23 Å). It is noteworthy that a chemically defined surface composed of alternating PMMA-rich and PS-rich stripes can be obtained at the micrometer scale in a lithography-free manner. In order to further scrutinize this adsorption phenomenon, a droplet of deionized water was directly placed on top of the surface with only PMMA adsorption layer (without PS-OH brush) and the three-phase contact line of water drop was observed with OM in reflection mode to visualize undulation of the contact line, which was mainly driven by different surface energies of hydrophilic native oxide and hydrophobic PMMA surface (Figure S1b). Further confirmation was made using XPS, revealing that a hydrocarbon peak (C-C and C-H) and a weak carboxylic peak were still maintained at a binding energy of 285.0 eV and at 288.8 eV before and after acetone washing, as displayed in Figure S1c. This strongly suggests that the chemical anchoring of the PMMA segments into the Si deposited with a very thin native oxide layer. This is in good accord with a previous study [7,46]. Subsequent surface treatment wth a PS-OH brush layer was conducted at 150 °C for 2 h triggered chemical anchoring of PS-OH onto a non-patterned Si surface (i.e., PS-rich surface) because of condensation reaction between hydroxyl groups of PS-OH and native oxide layer (Figure 2b,c) [47]. This treatment benefited the combined assemblies of preferential segregation of the PS-*b*-PDMS thin film from PMMA-rich regions to PS-rich regions (i.e., surface-tension-driven dewetting of di-BCP from physically adsorbed surfaces) at the micrometer scale and the solvent-vapor-mediated hierarchical structuring of the laying-down cylinders of the PDMS domains at the nanometer scale. Indeed, the Si right after Piranha cleaning, PMMA-adsorbed surface, and PS-OH brush-treated surface displayed different contact angles (CA) of 20.08°, 65.43°, and 85.31°, respectively (Figure S2). The PS-*b*-PDMS toluene solution with concentration of 10 mg mL^-1^ was spin-coated on top of the chemically defined gradient surface (Figure 2d) and then annealed for microphase-separation with toluene vapor. Over large areas of the PS-rich stripes with a width gradient, thin films PS-*b*-PDMS copolymer with high χ (~0.26; several times larger than polystyrene-*block*-poly (methyl methacrylate) (PS-*b*-PMMA)) were thermodynamically self-assembled into hierarchically ordered nanostructures [48,49]. BCPs with high χ are often suffering from slow self-assembly kinetics, which is highly expected with exponential decrease of the chain mobility [48,49]. 

**Figure 3:**
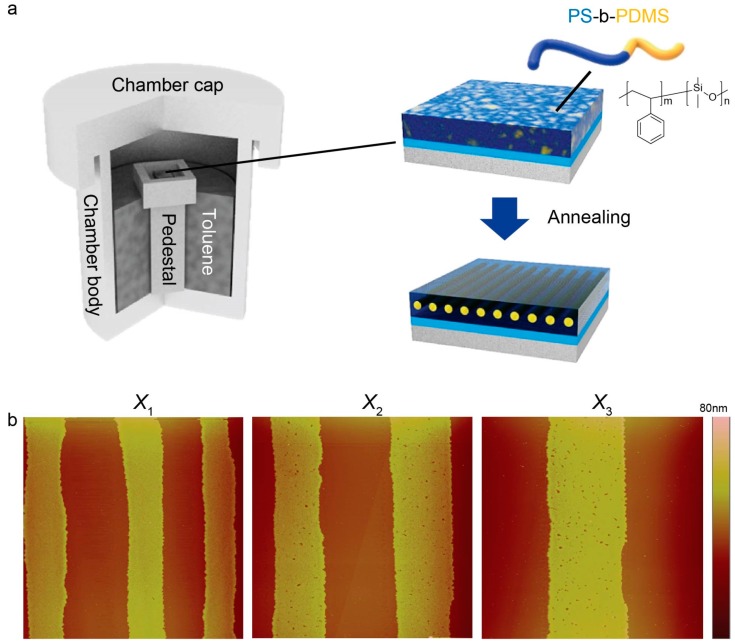
(**a**) Schematic description of the experimental setup for the solvo-thermal annealing; (**b**) representative atomic force microscopy (AFM) height images captured in three different regions: the innermost region (*X*_1_), the intermediate region (*X*_2_), and the outermost region (*X*_3_). Scan size was fixed at 40 × 40 μm^2^ for all three regions.

Thus, for such kinds of BCPs, solvent vapor annealing can be more commonly used for achieving better chain flexibility, thereby securing control over the morphological orientation of each domain. In more detail of this self-assembly, as exposure of the PS-*b*-PDMS thin film to toluene vapor proceeded with elapsed time (Figure 3a), the initially continuous PS-*b*-PDMS thin film dewetted from the PMMA-rich regions and then preferentially moved toward the PS-rich regions (Figure 2e). The atomic force microscopy (AFM) measurements showed that dimensions of the PS-rich equivalent to the center-to-center spacing minus stripe width (i.e., *λ*_c-c_‒*w*) gradually decreased with an increasing proximity to the wedge/Si contact center, ranging from 5.2 ± 0.2 μm at the innermost region, *X*_1_, to 7.1 ± 0.2 μm at the intermediate region, *X*_2_, and to 8.9 ± 0.3 μm at the outermost region, *X*_3_, respectively (Figure 3b). After the whole solvent-vapor annealing completed, thickness of the PS-*b*-PDMS thin film was found to be 25 nm in average, which was approximately commensurate with the periodic length scale of the domain spacing (≈ 32 nm) (Figure 3b).

Turning our attention to preferential segregation of di-BCP thin film from PMMA-rich regions to PS-rich regions during the SVA process could yield consideration of the surface energies (γ) and the effective *Hamaker* ($A_{\mathrm{eff}}$) constant for the van der Waals (vdW) interaction [50]. The PDMS has an even lower surface tension ($\gamma_{\mathrm{PDMS}}$ = 19.8 mN m^−1^) compared to PS ($\gamma_{\mathrm{PS}}$ = 40.7 mN m^−1^) at 20 °C [51], leading to its preferential segregation to the air surface; the PS tends to readily show affinity with PS-OH brush treated surface. Otherwise, in the PMMA-rich regions, the PS tends to dewet because of incompatibility between PS and PMMA that can be firmly understood by taking into account a excess intermolecular interaction free energy ($∆G_{t}$) composed of antagonistic (attractive/repulsive) long-range dispersion and short-range polar interactions, $∆G_{D}\left(T\right)$ and $∆G_{P}\left(T\right)$, defined as follows [52,53,54,55,56]:

(1) $$∆G_{t}(H)\;=\;∆G_{D}(H)+∆G_{P}(H)\;=\;-\frac{A_{132}}{{12\pi T}^{2}}{\;+\;S}_{p}\exp\left(-\frac{T}{l}\right),$$

where H is the PS-*b*-PDMS film thickness, $A_{132}$ is the effective *Hamaker* constant for media 1 and 2 interacting across medium 3 (i.e., 1: surface, 2: medium, 3: film), $S_{p}$ is the polar component of spreading coefficient related to short-range polar interaction, and l is a correlation length (~2.78 nm), respectively. Based on the above equation, destabilization of the polymeric thin films can be categorized into three modes: mode I ($A_{\mathrm{eff}\;}>\;0$, i.e., long-range attraction, and $S_{p}\;<\;0$, i.e., short-range attraction), mode II ($A_{\mathrm{eff}\;}>\;0$ and $S_{p}\;>\;0$, i.e., short-range repulsion), mode III ($A_{\mathrm{eff}}\;<\;0$, i.e., long-range repulsion, and $S_{p}\;<\;0$), and mode IV ($A_{\mathrm{eff}}\;<\;0$ and $S_{p}\;>\;0$). Based on this theoretical aspect, the case in our study can be verified by numerically calculating $A_{132}$ and $S_{p}$ using the following equations [52,53,54,55,56].

(2) $$A_{132}\;\approx \;\left(\sqrt{A_{11}}\;-\;\sqrt{A_{33}}\right)\left(\sqrt{A_{22}}\;-\;\sqrt{A_{33}}\right)\;\approx {\;24\pi d}_{0}^{2}\left(\sqrt{\gamma_{1}^{d}}\;-\;\sqrt{\gamma_{3}^{d}}\right)\left(\sqrt{\gamma_{2}^{d}}\;-\;\sqrt{\gamma_{3}^{d}}\right)$$

Here, $d_{0}$ and $\gamma_{i}^{d}$ indicate an atomic cutoff length (~0.158 nm) and the dispersive component of individual surface tensions, respectively. 

For the system consisting of the PMMA-rich surface (i.e., physically adsorbed PMMA surfaces) (media 1), air saturated with toluene vapor (media 2), and the PS and the PDMS (medium 3; the PS majority and PDMS minority of the PS-*b*-PDMS film), the values of $A_{\mathrm{PMMA}/\mathrm{Toluene}/\mathrm{PS}}$ and $A_{\mathrm{PMMA}/\mathrm{Toluene}/\mathrm{PDMS}}$ were calculated to be −1.68 × 10^−20^ and 3.20 × 10^−18^ J ($\gamma_{\mathrm{PMMA}}^{d}$ = 23.00, $\gamma_{\mathrm{Toluene}}^{d}$ = 26.95, $\gamma_{\mathrm{PS}}^{d}$ = 26.70, and $\gamma_{\mathrm{PDMS}}^{d}$ = 13.5 mN m^−1^). Using the equation (3), the polar components of the spreading coefficients for toluene/PS and toluene/PDMS were calculated to be 0.018 and −3.41 mN m^−1^, respectively [52,53,54,55,56].

(3) $$S_{p\;}{\;=\;\gamma }_{23}\left(\cos\theta \;-\;1\right)\;-{\;A}_{132}/{12\pi d}_{0}^{2}\;\left(∵\gamma_{23}={\left(\sqrt{\gamma_{2}}-\sqrt{\gamma_{3}}\right)}^{2}\right)$$

The modes IV and I can apply for the cases of PMMA/toluene/PS and PMMA/toluene/PDMS, respectively. The former represents long- and short-range repulsion, whereas the latter is predominantly governed by long- and short-range attraction. Since the volume fraction of PS is two times higher than PDMS, the mode IV can be considered to be primary in our study (Figure 3b). 

**Figure 4:**
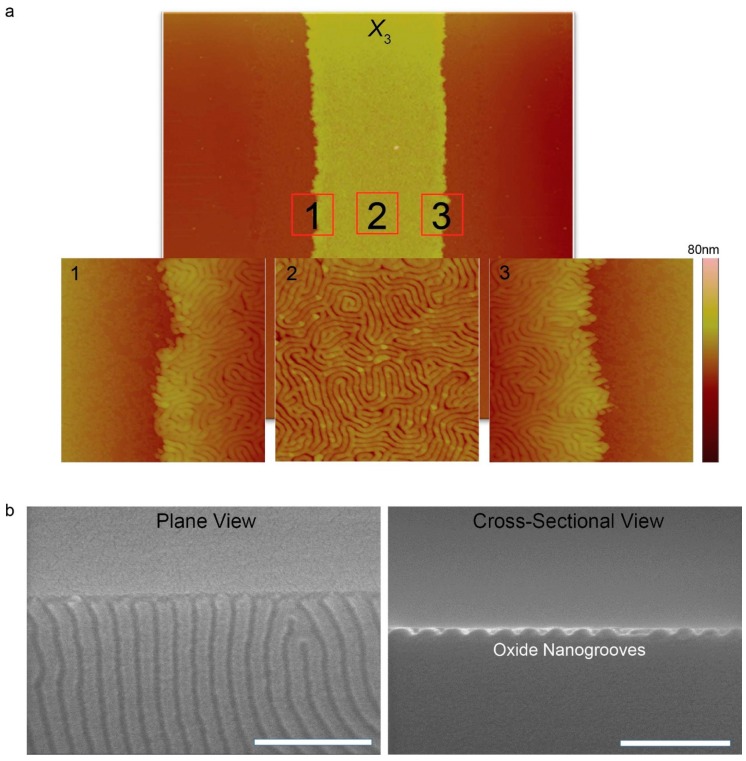
(**a**) Close-up AFM phase images (image size = 2 × 2 μm^2^) corresponding to the solid red-colored squares marked in 1, 2, and 3 in a AFM height image of the outermost region (*X*_3_); (**b**) FE-SEM images with scale bars of 200 nm (left: plane view and right: cross-sectional view) oxide nanogroove structures created through CF_4_ plasma etching and O_2_ plasma treatment.

Remarkably, in harmony with the preferential segregation of the PS-*b*-PDMS film toward the PS-rich region at the micrometer scale as demonstrated above, the spatial reconstruction of the PS-*b*-PDMS driven was carried out via spontaneous microphase separation driven by annealing with toluene vapor for 30 min at 35 °C. Such a spatial rearrangement of PS and PDMS nanodomains can be readily rationalized by the fact that toluene vapor is likely to swell more preferentially the PS components compared to the PDMS, thus triggering PS chains much more mobile. Indeed, toluene has different levels of affinity for PS and PDMS because of different polymer–solvent interaction parameters, χ, ($\chi_{\mathrm{PS}/\mathrm{Toluene}}$ = 0.441 and $\chi_{\mathrm{PDMS}/\mathrm{Toluene}}$ = 0.465) [51]. In addition, the preferential swelling of the PS blocks in response to toluene vapor can also be confirmed by enthalpy-derived interaction parameter ($\chi_{H}^{\infty }$) between solvent and solute molecules, which is well-defined as follows [57]:

(4) $$\chi_{H}^{\infty }\;=\;\left(\frac{V_{\mathrm{solvent}}}{\mathrm{RT}}\right){\left(\delta_{\mathrm{solvent}}\;-{\;\delta }_{\mathrm{solute}}\right)}^{2},$$

where $V_{\mathrm{solvent}}$, R, T, and δ represent the molar volume (cm^3^·mol^−1^), the gas constant (~1.987 cal·K^−1^·mol^−1^), temperature (K), and Hildebrand solubility parameter ((cal·cm^−3^)^1/2^), respectively. Based on the Hildebrand solubility parameters of PS (≈ 9.04), PDMS (≈ 7.58), and toluene (≈ 8.91) [57], the values of $\chi_{H}^{\infty }$ between PS- and PDMS-toluene vapor are calculated to be 0.00 and 0.035, respectively, thus leading to preferential swelling of the PS blocks. Use of the 35 °C slightly higher than room temperature is strongly motivated for compensating slow self-assembly kinetics of BCPs with high χ, thus accelerating speed of microphase separation. A previous study experimentally well-described effect of annealing temperature on morphological evolution of the PS-*b*-PDMS, confirming that defect density of spatially rearranged nanodomains gradually decreased as the solvo-thermal annealing temperature increased up to 85 °C [58]. In our experiment, 35 °C was selected as the solvo-thermal annealing temperature on behalf of 85 °C in order to induce the destabilization of the PS-*b*-PDMS thin film at a slow, steady rate. The close-up AFM phase images clearly show hierarchically ordered nanostructures of high χ BCP in the chemically defined striped region (Figure 4). 

Lateral mean size of the laying down PDMS cylinders was found to be 16 nm, aligned parallel to the PS-rich surface. Since the PS-rich regions (i.e., PS-OH treated areas) are much wider than the lateral dimension of the laying down PDMS cylinders, big confinement effect was not observed. However, if the keys to fine tuning the CESA process are firmly secured, dimensional scale of the evaporatively self-assembled patterns would be reduced, thus enhancing probability of confinement effect. Conversion of PDMS to SiO_x_ is mediated by atomic oxygen derived from the photo-cleavage of ozone. All the samples were etched by CF_4_ plasma followed by O_2_ plasma treatment to produce the oxidized nanogroove structures by a reactive ion etching (RIE) system as clearly shown in Figure 4b,c. It is worthy to note that these oxidized nanogroove structures are not permanent because of hydrophobic recovery driven by the gradual coverage of a SiO_x_ structure with free siloxane linkages (≡S‒O‒S≡) and reorientation of polar hydroxyl groups (‒OH) [59,60].

## 4. Conclusions

In summary, we demonstrated a simple and facile approach to crafting hierarchically ordered nanostructures via two consecutive self-assembly processes thermodynamically driven at the micro- and nanometer scale. Notably, polymer structures produced by CESA served as passivation patterns for additional surface treatment and the resulting alternating surfaces with different surface energies enabled self-assembly of high χ BCP in energetically favorable regions during solvent-vapor annealing. The strategy exploited in the present study requires no need for costly and multistep lithography techniques. The spatially arranged nanostructures with hierarchies may seek potential applications in such research fields as optics, electronics, sensors, and energy harvesting devices. Furthermore, they can also serve as an effective platform for preparing three-dimensionally stacked nanostructures, selectively coordinating metallic nanoparticles, and templating for cell adhesion and motility.

## Supplementary Materials

The following are available online at https://www.mdpi.com/1996-1944/13/2/304/s1, Figure S1: OM observation and XPS investigation of PMMA adsorption on to SiO_x_ surface, Figure S2: Contact angle measurements of different surfaces.
