# Supplementary material for: Lithography-Free Route to Hierarchical Structuring of High-χ Block Copolymers on a Gradient Patterned Surface
Source: Materials (Basel). 2020 Jan 9;13(2):304. doi: 10.3390/ma13020304 (PMC7013446; doi:10.3390/ma13020304)
Supplement: Supplementary file 1 [file materials-13-00304-s001.pdf]

materials

Supplementary

Lithography-Free Route to Hierarchical Structuring of High-χ Block Copolymers on a Gradient Patterned Surface

Ha Ryeong Cho[1,†], Ayoung Choe[2,†], Woon Ik Park[3], Hyunhyub Ko[2,*], Myunghwan Byun[1,4,*]

[1] Department of Materials Engineering, Keimyung University, Daegu 42601, Republic of Korea 1; haryeungcho@gmail.com
[2] School of Energy and Chemical Engineering, Ulsan National Institute of Science and Technology (UNIST), Ulsan 44919, Republic of Korea 2; ayoung5678@unist.ac.kr, hyunhko@unist.ac.kr
[3] Department of Materials Science and Engineering, Pukyong National University, Pusan 48513, Republic of Korea 3; thane0428@pknu.ac.kr
[4] Department of Advanced Materials Engineering, Keimyung University, Daegu 42601, Republic of Korea 4; myunghbyun@kmu.ac.kr
† These authors contributed equally to this work
* Correspondence: hyunhko@unist.ac.kr; Tel.: +82-52-217-2532 (H.K.), myunghbyun@kmu.ac.kr; Tel.: +82-53-580-5228 (M.B.)

Received: date; Accepted: date; Published: date

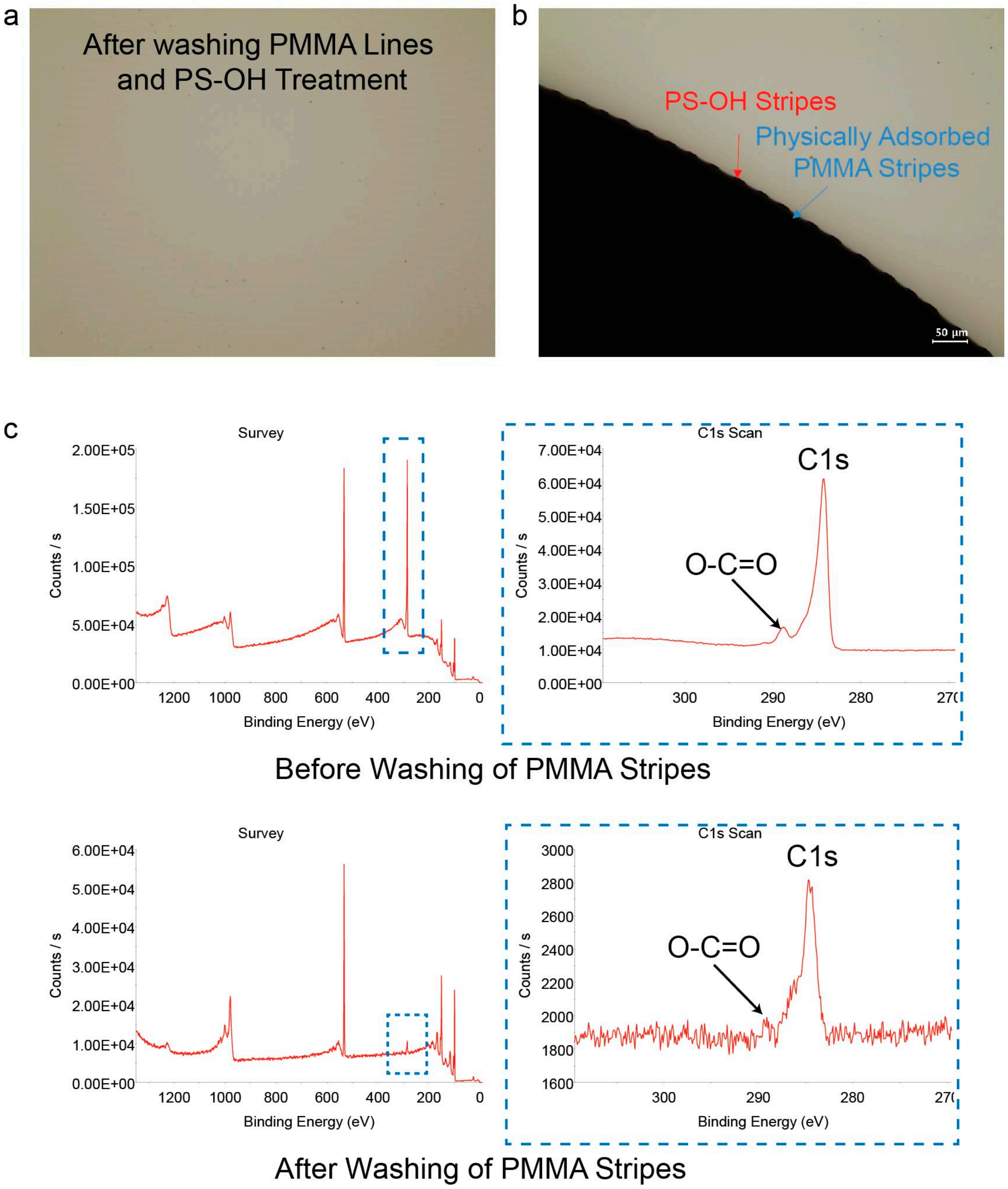

Figure S1. OM observation and XPS investigation of PMMA adsorption on to SiO_x surface.

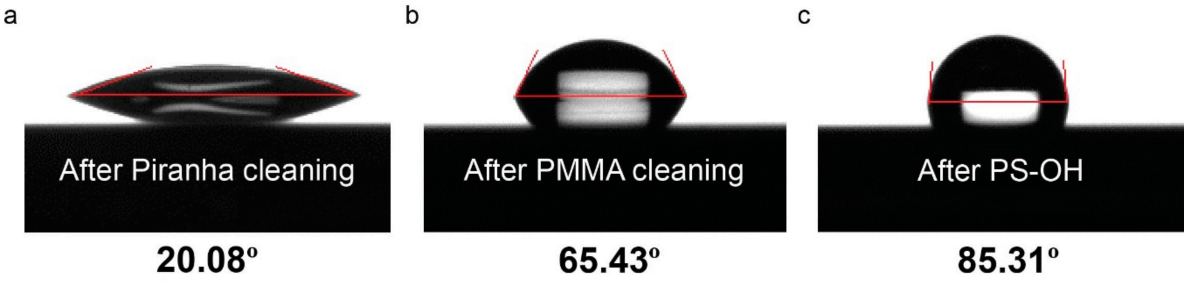

Figure S2: Contact angle measurements of different surfaces.
